# A bibliometric analysis of T cell and atherosclerosis

**DOI:** 10.3389/fimmu.2022.948314

**Published:** 2022-10-13

**Authors:** Namin Wei, Yan Xu, Ya’nan Li, Jingjing Shi, Xuesong Zhang, Yaping You, Qianqian Sun, Huaqiang Zhai, Yuanhui Hu

**Affiliations:** ^1^ School of Chinese Materia Medica, Beijing University of Chinese Medicine, Beijing, China; ^2^ Department of Cardiovascular Diseases, Guang’anmen Hospital, China Academy of Chinese Medical Sciences, Beijing, China

**Keywords:** T cell, atherosclerosis, CiteSpace, VOSviewer, bibliometrics, visualization

## Abstract

Atherosclerosis (AS) is widespread and develops into circulatory system problems. T cells play an essential regulatory role in AS occurrence and development. So far, there is no bibliometric research on T cells and AS. To learn more about T cell and AS development, 4,381 records were retrieved from Web of Science™ Core Collection. Then, these records were scientometrically analyzed using CiteSpace and VOSviewer in terms of spatiotemporal distribution, author distribution, subject categories, topic distribution, references, and keywords. Our analysis provides basic information on research in the field, demonstrates that the field has stabilized over the past decade, and identifies potential partners for interested researchers. Current research hotspots in this field mainly include the inflammatory mechanism, immune mechanism, related diseases, and related cytokines of AS. B cell, mortality, inhibition, and monocyte represent the frontiers of research in this field, undergoing an explosive phase. We hope that this work will provide new ideas for advancing the scientific research and clinical application of T cell and AS.

## 1 Introduction

Atherosclerosis (AS) is a chronic inflammatory disease with an autoimmune component that is regulated by innate and adaptive immunity (1–3). AS exists widely in countries all over the world and progresses into a circulatory system problem (4, 5). It is estimated that by 2030, more than 23 million people will die annually from AS (6). At present, the treatment of AS has been expanded from simple lipid-lowering, lipid-regulating and plaque stabilization to the prevention and control of immune damage and inflammation (2).

Studies have shown that atherosclerotic lesions contain a large number of immune cells, especially monocyte macrophages and T cells (7). Among them, the key to adaptive immune regulation lies in the regulation of T cells. T cells are activated in both cellular immunity and humoral immunity, and different T cell subtypes play different roles (8). Examples, Th1 cells secrete pro-inflammatory cytokines such as interferon (IFN) -γ and interleukin (IL) -12 to promote monocyte infiltration and macrophage activation, thus promoting AS (9). Th2 cells, secrete IL-4, IL-5, IL-10, and IL-13, and their anti-atherosclerotic effects remain controversial (10, 11). Regulatory T cells (Tregs) are immunosuppressive cells, which play a protective role in AS by inhibiting Th1/Th17 mediated pro-inflammatory response and down-regulating Dendritic Cell Presenting Antigen (12–14).

Bibliometrics is a branch of informatics that takes the literature system and bibliometric characteristics as the research object and conducts quantitative and qualitative analyses of literature. This method can quantitatively measure the contour distribution, relationship, and clustering of the research field (15) and has become one of the prevalent techniques for assessing academic work credibility, quality, and impact (16, 17). Specifically, the evaluation can comprise contributions and influence of different authors, countries/regions, institutions, disciplines, and journals, as well as assess the status, trends, and frontiers of research activities (18–20). VOSviewer and CiteSpace are commonly used bibliometric visualization tools for data analysis and visualization (21, 22). They are effective methods for evaluating the thematic development of structured content and can facilitate intuitive comprehension among readers (23).

Although this type of literature has been widely utilized in other fields in recent years, to our knowledge, so far, there is no bibliometric study in the field of T cell and AS. To fill this knowledge gap, this study is based on the Web of Science™ Core Collection (WoSCC). Relevant bibliometric data (annual articles, countries/regions, authors, institutions, disciplines, journals, references and keywords) for each T cell and AS research field were retrieved, and descriptive statistics were performed. This paper discusses the research status, hotspots, and frontiers of literature on T cell and AS and draws knowledge maps using CiteSpace and VOSviewer to serve as a reference for relevant future research.

## 2 Methods

### 2.1 Data collection

Web of Science is the primary research platform for hard science, social sciences, arts, and humanities information, as well as the independent global citation database of the world’s most trusted publishers (24). To improve data representativeness and accessibility, we retrieved WoSCC. The following search terms were employed to retrieve literature from WoSCC regardless of language or document type: Topic = (“T-lymphocyte” OR “T Lymphocytes” OR “T-Lymphocyte” OR “Thymus-Dependent Lymphocytes” OR “Lymphocyte, Thymus-Dependent” OR “Lymphocytes, Thymus-Dependent” OR “Thymus Dependent Lymphocytes” OR “Thymus-Dependent Lymphocyte” OR “T-Cells” OR “T-Cell” OR “T Cell” OR “Cell, T” OR “Cells, T” OR “T Cells” OR “T Lymphocyte” OR “Lymphocyte, T” OR “Lymphocytes, T”) AND (“Atherosclerosis” OR “Atheroscleroses” OR “Atherogenesis”). The retrieved data were collected on February 20, 2022, to avoid any potential deviation due to daily updates. A total of 4,381 records comprising ten document types were obtained. Notably, 3,096 original research papers accounted for 70.669% of total records (**Table 1**). Review articles ranked second (n=978), accounting for 22.324% of the total records. The other eight document types met abstracts (n=184), editorial materials (n=98), proceedings papers (n=65), letters (n=21), book chapters (n=19), early access (n=18), corrections (n=4), and retracted publications (n=1). A total of 4,381 records were exported in the form of full records with cited references, saved as plain text files, and stored in download_.txt format. A total of 199,057 times cited, 122,704 citing articles, 181 h-index, and 45.440 average per item were obtained.

**Table 1:** Document types of the publications.

No.	Document Types	Record Count	Citing Articles	Times Cited	Average per item	% Of 4,381	H-index
1	Articles	3,096	76,6300	120,367	38.88	70.669	142
2	Review Articles	978	61,387	77,394	79.13	22.324	125
3	Meeting Abstracts	184	60	41	0.22	4.200	3
4	Editorial Materials	98	1,102	1,127	11.50	2.237	18
5	Proceedings Papers	65	3,857	3,941	60.63	1.484	34
6	Letters	21	118	119	5.67	0.479	7
7	Book Chapters	19	3,372	3,443	181.21	0.434	15
8	Early Access	18	18	18	1.00	0.411	2
9	Corrections	4	9	9	2.25	0.091	1
10	Retracted Publications	1	1	1	0.33	0.023	1

### 2.2 Data analysis

**Figure 1:**
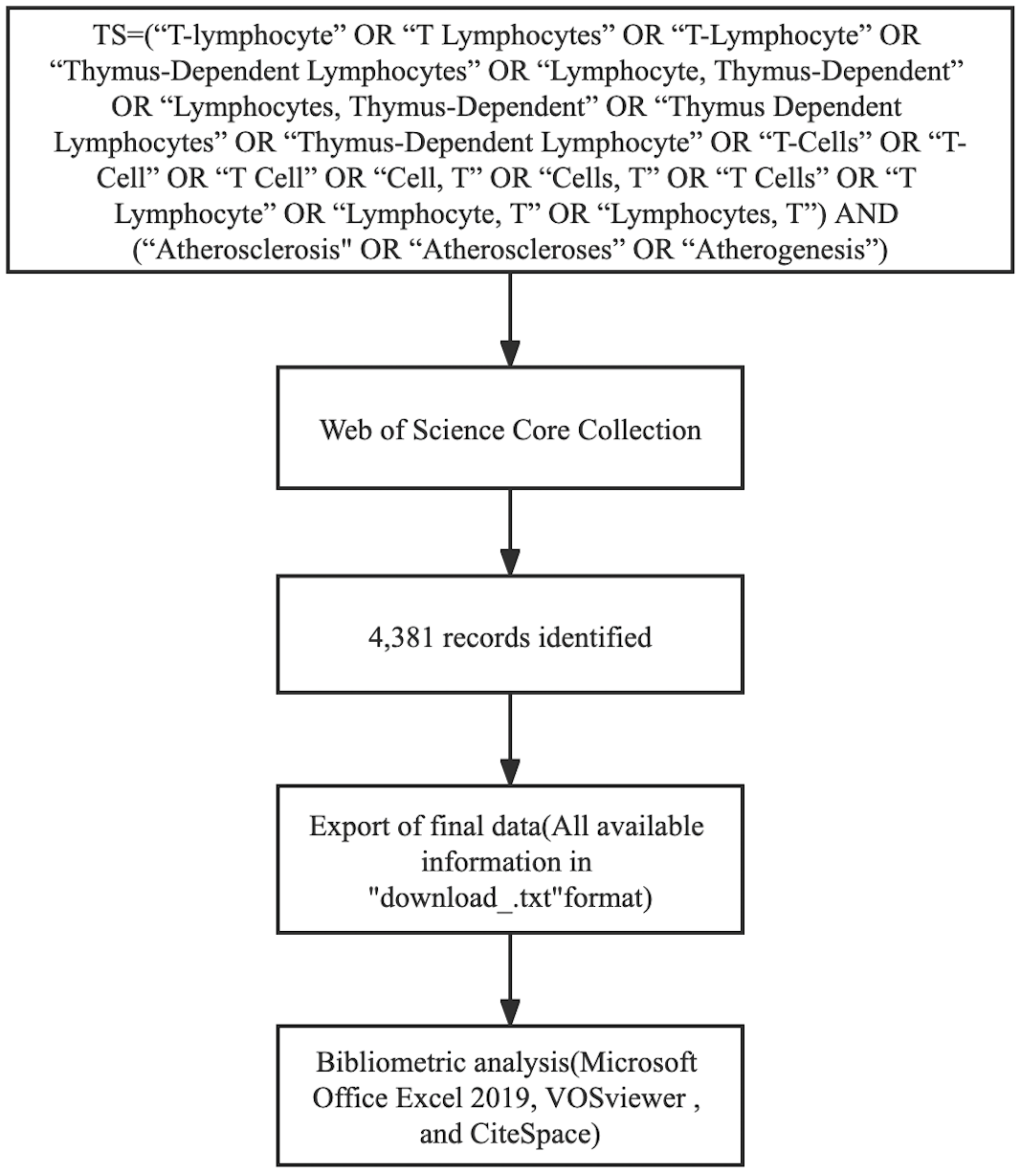
The flowchart illustrating the search strategy and selection process in T cell and AS.

The Microsoft Office Excel 2019, VOSviewer (v.1.6.17), and CiteSpace (v.6.1.R3 Advanced) analyzed all 4,381 documents. VOSviewer (a bibliometric software) is a Java-based free software developed by Van Eck and Waltman of the center for science and technology studies (CWTS) of Leiden University in the Netherlands in 2009. It has a strong graphic ability and is suitable for processing large-scale data (22). CiteSpace was developed by Professor Chen Chaomei of Drexel University in the United States. It is a document visualization analysis software gradually developed for bibliometrics analyses and data visualization (21, 25). CiteSpace was deployed to visually display the basic knowledge and hotspots of T cell and AS and predict its research frontiers (26). The emergence and development of these two software have strongly promoted the research and application expansion of the entire information visualization field (27). **Figure 1** shows the flowchart for search strategy and selection process in this study.

## 3 Results

### 3.1 Temporal distribution map of the literature

**Figure 2:**
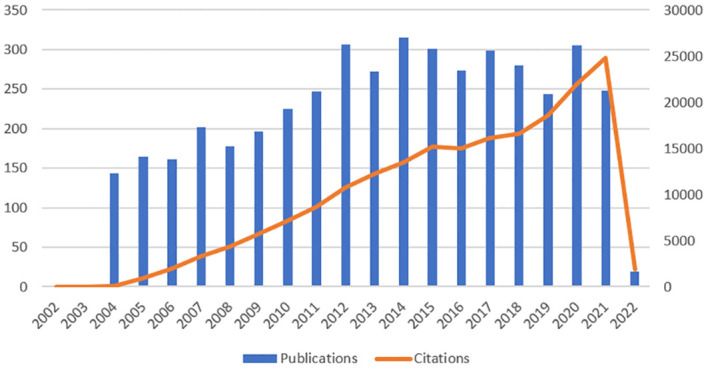
Trends in the growth of publications and the number of citations.

The number of publications over a period can reflect the research speed and trend in this field (28). As depicted in **Figure 2**, there have been publications in this field since 2004, demonstrating that it has garnered interest. Since 2007, the number of publications has basically remained constant at 200. In 2014, 315 papers were published. In recent ten years, the number of articles published has been about 250, indicating that the field is relatively stable. Citations are growing rapidly and increasing annually.

### 3.2 Distribution of countries/regions

As revealed in **Table 2**, the largest number of published literature was from USA, China, and Germany, respectively, accounting for 32.938%, 15.156%, and 11.048% of the total reports. The total number of studies from the three countries accounted for more than half of the total reports, implying a high interest in research in T cell and AS. Studies from Sweden (96.390), USA (68.690), and Australia (60.770) had the highest average per item, indicating that the three countries started research on T cell and AS, and their results are relatively mature. Among the top 10 countries/regions in **Table 2**, the centrality of five countries is greater than or equal to 0.1. They are USA (0.61), Germany (0.19), England (0.19), China (0.14), and France (0.10), indicating that these countries play a crucial role in the research in this field.

**Table 2:** Top 10 productive countries/regions in T cell and AS.

Rank	Countries/Regions	Record Count	% Of 4,381	Average Per Item	H-index	Citations	Total link strength	Centrality
1	USA	1,443	32.938	68.69	146	98,818	871	0.61
2	China	664	15.156	21.49	54	14,267	225	0.14
3	Germany	484	11.048	55.95	81	27,070	542	0.19
4	Netherlands	352	8.035	50.64	72	17,824	414	0.06
5	Sweden	300	6.848	96.39	67	28,886	258	0.02
6	England	292	6.665	42.50	63	12,387	395	0.19
7	Italy	279	6.368	40.34	56	11,254	258	0.08
8	Japan	262	5.980	40.08	53	10,489	133	0.02
9	France	233	5.318	49.18	58	11,459	252	0.10
10	Australia	149	3.401	60.77	48	8,965	128	0.05

Here includes the automatically calculated total link strength by VOSviewer.

**Figure 3:**
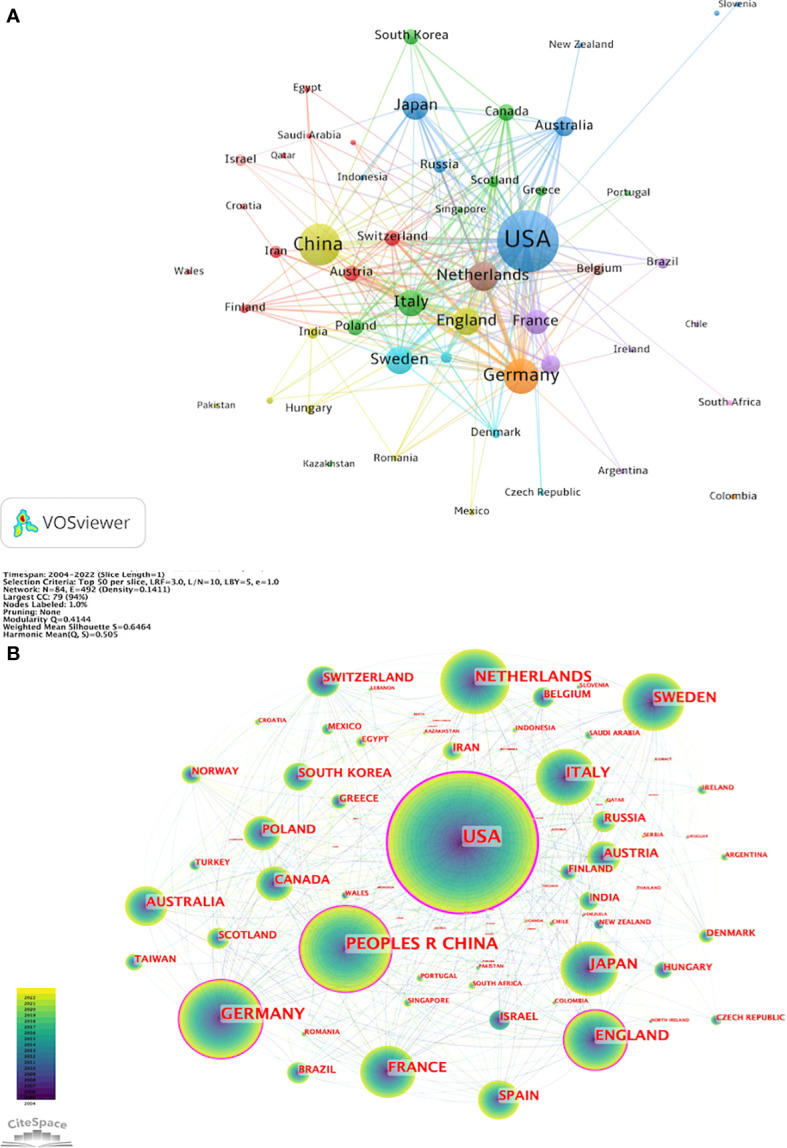
Cooperation map of countries/regions in T cell and AS. **(A)** A visual map for VOSviewer network. **(B)** A visual map for CiteSpace network.

VOSviewer parameters were set as follows: Method (Linlog/modularity) and a minimum number of country documents: 5. The obtained results were retrieved from 85 countries, with 51 meeting the thresholds. Based on co-authorship analysis, VOSviewer classifies countries into different clusters. Nodes with different colors in the figure represent different clusters. The size of nodes represents the number of documents, and the thickness of lines represents the number of connections between nodes. **Figure 3** displays the cooperation map of countries/regions in T cell and AS. **Figure 3A** shows that USA largely cooperated with Australia, Indonesia, Japan, New Zealand, Russia, Serbia, and Slovenia; China closely cooperated with England, Hungary, India, Mexico, Pakistan, Romania, and Turkey; Austria frequently cooperated with Croatia, Egypt, Finland, Iran, Lebanon, Qatar, Saudi Arabia, Switzerland, and Wales. Italy closely cooperated with Canada, Greece, Kazakhstan, Poland, Portugal, Scotland, Singapore, and South Korea. CiteSpace parameters were set as follows: time slice (2004–2022), years per slice (1), term source (entire selection), node type (country), and selection criteria (top N=50). Other parameters were left at the default settings. The purple-circled nodes in **Figure 3B** represent countries with high centrality. CiteSpace employs this indicator to measure the importance of countries in this field. From 2004 to 2022, the color changes from purple to yellow.

### 3.3 Distribution of authors and research institutions

Hansson GK from the Department of Medicine, Center for Molecular Medicine (G.K.H.) was the author with the most published articles, followed by Weber C from the Institute for Cardiovascular Prevention (IPEK), Ludwig Maximilians University and German Centre for Cardiovascular Research (DZHK) and Nilsson J from Department of Clinical Sciences Malmö, Lund University, Sweden (**Table 3**).

**Table 3:** Top 10 authors in T cell and AS.

Rank	Author	Record Count	% Of 4,381	Affiliations	Average Per Item	H-index	Centrality
1	Hansson GK	97	2.214	Department of Medicine, Center for Molecular Medicine (G.K.H.).	213.81	49	0.01
2	Weber C	78	1.780	Institute for Cardiovascular Prevention (IPEK), Ludwig Maximilians University, Pettenkoferstraße 8a & 9, Munich, Germany; German Centre for Cardiovascular Research (DZHK), partner site Munich Heart Alliance, Pettenkoferstraße 8a & 9, Munich, Germany.	75.85	37	0.01
3	Nilsson J	70	1.598	Department of Clinical Sciences Malmö, Lund University, Sweden.	30.11	29	0.01
4	Mallat Z	69	1.575	Division of Cardiovascular Medicine, Department of Medicine, Box 157 Addenbrooke’s Hospital, Hills Road, Cambridge, CB2 0QQ, UK; Institut National de la Santé et de la Recherche Médicale (Inserm), Unit 970, Paris Cardiovascular Research Center, Paris, France.	64.67	33	0.01
5	Kuiper J	68	1.552	Division of BioTherapeutics, Leiden Academic Centre for Drug Research, Leiden University, Einsteinweg 55, Room EE1.17, 2333 CC Leiden, the Netherlands.	34.28	26	0.01
6	Lutgens E	68	1.552	Department of Medical Biochemistry, Amsterdam Cardiovascular Sciences (ACS), Amsterdam University Medical Centers, University of Amsterdam, 1105AZ Amsterdam, The Netherlands; Institute for Cardiovascular Prevention (IPEK), Ludwig Maximilian’s University, 80336 Munich, Germany; German Centre for Cardiovascular Research (DZHK), Partner Site Munich Heart Alliance, 80802 Munich, Germany.	30.81	23	0.01
7	Libby P	57	1.301	Division of Cardiovascular Medicine, Brigham and Women’s Hospital, Harvard Medical School, Boston, Mass 02115, USA.	231.44	41	0.02
8	Ley K	53	1.210	Department of Biomedical Engineering, University of Virginia, Health Sciences Center, Charlottesville, VA 22908, USA.	73.45	27	0.00
9	Tedgui A	51	1.164	Paris Cardiovascular Research Center-PARCC, Université de Paris, INSERM UMR-S 970, 75012 Paris, France.	74.80	27	0.00
10	Gerdes N	45	1.027	Institute for Cardiovascular Prevention (IPEK), Ludwig Maximilians University, Pettenkoferstraße 8a & 9, Munich, Germany; Division of Cardiology, Pulmonology, and Vascular Medicine, Medical Faculty, University Hospital Düsseldorf, Moorenstraße 5m 0225 Düsseldorf, Germany.	31.93	18	0.01

**Figure 4:**
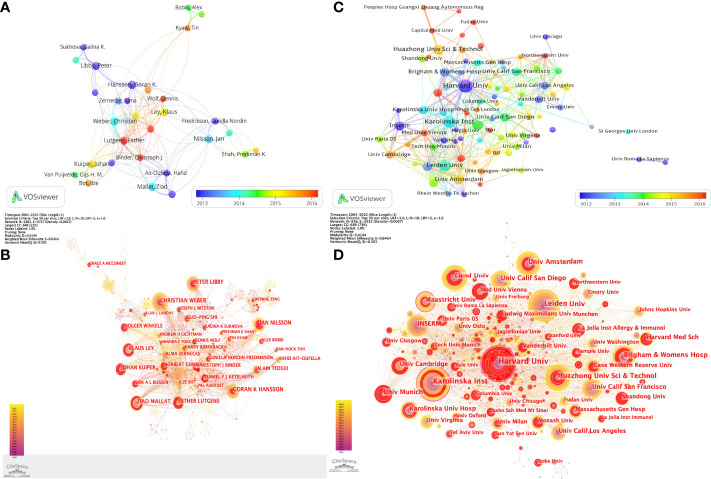
Collaboration networks among authors and among institutions in T cell and AS. **(A)** A visual map for VOSviewer network among authors. **(B)** A visual map for CiteSpace network among authors. **(C)** A visual map for VOSviewer network among institutions. **(D)** A visual map for CiteSpace network among institutions.

VOSviewer parameters were set as follows: Method (Linlog/modularity) and a minimum number of documents of an author: 20. The obtained results were retrieved for 21,108 authors, and 35 met the thresholds. Based on co-authorship analysis, VOSviewer classifies authors into different clusters and colors them according to the time course in which they appear, superimposing time on the network of co-occurrence authors. Each node in **Figure 4A** represents each author, the size of the circle reflects the number of articles published by researchers, and the line connecting the circles represents the co-occurrence relationship between authors. As revealed in **Figure 4A**, different clusters represent cooperation among authors. Aukrust, Pal., Hansson, Goran K., Ketelhuth, Daniel F. J., Libby, Peter, Lichtman, Andrew H., Shi, Guo-Ping, Sukhova, and Galina K closely cooperated. Gerdes, Norbert, worked closely with Ley, Klaus, Lutgens, Esther, Weber, Christian, Winkels, Holger, Wolf, Dennis, Zernecke, Alma. Bjorkbacka, Harry, Chyu, Kuang-Yuh, Fredrikson, Gunilla Nordin, Nilsson, Jan, Shah, and Prediman K closely cooperated. Biessen, Erik A. L., worked closely with Bot, Ilze, Kuiper, Johan, Van Puijvelde, and Gijs H. M. Ait-Oufella, worked closely with Hafid, Binder, Christoph J., Mallat, Ziad, Tedgui, Alain. Bobik, and Alex, worked closely with Kyaw, Tin, Toh, and Ban-Hock. CiteSpace parameters were set as follows: time slice (2004–2022), years per slice (1), term source (entire selection), node type (author), and selection criteria (top N=50). Other parameters were left at the default settings. **Figure 4B** shows that a visual map of authors for CiteSpace network. The co-citation relationship is indicated by the line connecting the nodes. The size of the node is determined by the citations. The colors represent different years. From 2004 to 2022, the color changes from purple to yellow.

As presented in **Table 4**, Harvard University (460 papers) was the institution that published the most research papers, followed by University of California System (413 papers) and Karolinska Institutet (318 papers). Karolinska Institutet is the research institution with the highest average per item (133.420). Harvard University closely follows it with an average per item of 115.670 and University of California System with an average per item of 62.850. Among the top 10 institutions in T cell and AS, there are three institutions whose centrality is greater than or equal to 0.10, namely Harvard University (0.13), Karolinska Institute (0.12) and Maastricht University (0.10).

**Table 4:** Top 10 institutions in T cell and AS.

Rank	Institution	Record Count	% Of 4,381	Citing Articles	Times Cited	Average per item	H-index	Centrality
1	Harvard University (USA)	460	10.500	22,851	27,993	115.67	81	0.13
2	University of California System (USA)	413	9.427	10,821	13,576	62.85	67	0.05
3	Karolinska Institutet (Sweden)	318	7.259	18,688	24,550	133.42	60	0.12
4	Leiden University (Netherlands)	285	6.505	4,229	4,994	42.32	37	0.07
5	Institut National de la Sante et de la Recher Che Medicale, Inserm (France)	197	4.497	7,554	9,826	49.88	53	0.09
6	University of Munich (Germany)	129	2.945	5,105	6,368	49.36	44	0.06
7	Universite de Paris (France)	109	2.488	4,132	5,293	48.56	37	0.02
8	University of Amsterdam (Netherlands)	108	2.465	3,503	4,029	37.31	36	0.06
9	Maastricht University (Netherlands)	105	2.397	4,492	5,304	50.51	41	0.10
10	Huazhong University of Science Technology (China)	93	2.123	2,283	2,876	30.92	27	0.04

VOSviewer parameters were set as Method (Linlog/modularity) and a minimum number of documents of an institution: 20. The obtained results were retrieved from 3,455 institutions, and 91 met the thresholds. As demonstrated in **Figure 4C**, seven clusters were formed, with close cooperation among representatives of the constituent institutions of each cluster. Cluster 1 consisted of the following institutions: Harvard Univ, Icahn Sch Med Mt Sinai, Kings Coll London, Massachusetts Gen Hosp, Baylor Coll Med, Brigham & Women’s Hosp, Capital Med Univ, Fudan Univ, Harvard Med Sch, Huazhong Univ Sci & Technol, Nanjing Med Univ, NHLBI, NIAID, Peking Univ, Peoples Hosp Guangxi Zhuang Autonomous Reg, Shandong Univ, Shanghai Jiao Tong Univ, Sun Yat-Sen Univ, Temple Univ, Univ Florida, Univ Michigan, and Wuhan Univ closely collaborated. Cluster 2 contained the following institutions: Albert Einstein Coll Med, Case Western Reserve Univ, Cedars Sinai Med Ctr, Columbia Univ, Emory Univ, Johns Hopkins Univ, Lund Univ, Mcgill Univ, Monash Univ, Northwestern Univ, Stanford Univ, Univ Alabama Birmingham, Univ Calif Los Angeles, Univ Calif San Francisco, Univ Chicago, Univ Minnesota, Univ Vermont, Univ Washington, and Vanderbilt Univ closely collaborated. Cluster 3 comprised the following institutions: Hannover Med Sch, Heidelberg Univ, Leiden Univ, Ludwig Maximilians Univ Munchen, Maastricht Univ, Rhein Westfal Th Aachen, Tech Univ Munich, Univ Amsterdam, Univ Groningen, Univ Maastricht, Univ Med Ctr Utrecht, Univ Munich, Univ Sao Paulo, Univ Ulm, and Yale Univ closely collaborated. Cluster 4 included the following institutions: Boston Univ, Ist Super Sanita, Nyu, St Georges Univ London, Tel Aviv Univ, Ucl, Univ Calif Davis, Univ Cattolica Sacro Cuore, Univ Milan, Univ New S Wales, Univ Penn, Univ Roma La Sapienza, Univ Sydney, and Univ Zurich closely collaborated. Cluster 5 consisted of the following institutions: Austrian Acad Sci, Inserm, Karolinska Inst, Karolinska Univ Hosp, Med Univ Vienna, Univ Cambridge, Univ Oslo, and Univ Paris closely collaborated. Cluster 6 contained the following institutions: La Jolla Inst Allergy & Immunol, La Jolla Inst Immunol, Univ Calif San Diego, Univ Freiburg, Univ Tokyo, and Univ Virginia closely collaborated. Cluster 7 comprised the following institutions: Jagiellonian Univ, Univ Glasgow, Univ Naples Federico Ii, and Univ Oxford closely collaborated. CiteSpace parameters were set as follows: time slice (2004–2022), years per slice (1), term source (entire selection), node type (institution), and selection criteria (top N=50). Other parameters were left at the default settings. **Figure 4D** shows that a visual map of institutions for CiteSpace network. Through the number of connections, we can see that the links between various institutions are relatively close. We can also clearly see that the nodes with purple outer circles and larger nodes are important participating institutions in T cell and AS.

### 3.4 Distribution of disciplines and journals

Regarding the number of published papers, Cardiac Cardiovascular Systems and Peripheral Vascular Disease and Immunology were the top three disciplines (**Table 5**). Additional subjects in the literature include Biochemistry Molecular Biology (18.535%), Hematology (11.276%), Cell Biology (10.454%), Medicine Research Experimental (9.792%), Pharmacology Pharmacy (7.784%), Multidisciplinary Sciences (7.464%), Endocrinology Metabolism (5.387%), Infectious Diseases (3.675%), and other subjects.

**Table 5:** Top 20 categories in T cell and AS.

Rank	Record Count	Web of Science Categories	% Of 4,381
1	967	Cardiac Cardiovascular Systems	22.073
2	922	Peripheral Vascular Disease	21.045
3	812	Immunology	18.535
4	494	Biochemistry Molecular Biology	11.276
5	458	Hematology	10.454
6	429	Cell Biology	9.792
7	341	Medicine Research Experimental	7.784
8	327	Pharmacology Pharmacy	7.464
9	236	Multidisciplinary Sciences	5.387
10	161	Endocrinology Metabolism	3.675
11	146	Infectious Diseases	3.333
12	136	Medicine General Internal	3.104
13	116	Physiology	2.648
14	94	Pathology	2.146
15	89	Microbiology	2.031
16	89	Rheumatology	2.031
17	73	Oncology	1.666
18	70	Surgery	1.598
19	67	Virology	1.529
20	62	Biophysics	1.415

As listed in **Table 6**, *Arteriosclerosis Thrombosis and Vascular Biology* was the journal with the highest number of publications in T cell and AS, followed by *Atherosclerosis* (179 publications), *Plos One* (139 publications), *Circulation* (131 publications), *Cardiovascular Research* (95 publications) and *Circulation Research* (95 publications). In co-citing journals, *Circulation* (39.918) was the journal with the highest impact factors (IF), followed by *European Heart Journal* (35.855), *Circulation Research* (23.213), and *Cardiovascular Research* (13.081). Among these journals, the IF of fifteen journals is greater than 10. Among them, the journal with the highest IF is *New England Journal of Medicine* (176.079), followed by *Nature Reviews Immunology* (108.555), *Nature Medicine* (87.241), *Nature* (69.504) and *Science* (63.741).

**Table 6:** Top 20 journals and co-cited journals in T cell and AS.

Rank	Record Count	% Of 4,381	Journal	IF (2021)	JCR	Co-cited Journal	Frequency	Degree	Centrality	IF (2021)	JCR
1	243	5.547	*Arteriosclerosis Thrombosis and Vascular Biology*	10.514	Q1/Q1	*Circulation*	3,178	30	0.04	39.918	Q1/Q1
2	179	4.086	*Atherosclerosis*	6.847	Q1/Q1	*Arteriosclerosis Thrombosis and Vascular Biology*	3,023	34	0.10	10.514	Q1/Q1
3	139	3.173	*Plos One*	3.752	Q2	*Journal of Immunology*	2,712	31	0.10	5.426	Q2
4	131	2.990	*Circulation*	39.918	Q1/Q1	*Journal of Clinical Investigation*	2,596	29	0.02	19.456	Q1
5	95	2.168	*Cardiovascular Research*	13.081	Q1	*Proceedings of the National Academy of Sciences of the United States of America*	2,399	28	0.01	12.779	Q1
6	95	2.168	*Circulation Research*	23.213	Q1/Q1/Q1	*Circulation Research*	2,377	27	0.03	23.213	Q1/Q1/Q1
7	72	1.643	*Journal of Immunology*	5.426	Q2	*Atherosclerosis*	2,361	30	0.03	6.847	Q1/Q1
8	71	1.621	*Frontiers in Immunology*	8.786	Q1	*Nature*	2,173	26	0.00	69.504	Q1
9	51	1.164	*European Heart Journal*	35.855	Q1	*New England Journal of Medicine*	2,130	28	0.06	176.079	Q1
10	47	1.073	*Thrombosis and Haemostasis*	6.681	Q1/Q1	*Journal of Experimental Medicine*	2,102	27	0.05	17.579	Q1/Q1
11	40	0.913	*International Journal of Molecular Sciences*	6.208	Q1/Q2	*Nature Medicine*	1,982	27	0.03	87.241	Q1/Q1/Q1
12	38	0.867	*Scientific Reports*	4.996	Q2	*Journal of Biological Chemistry*	1,816	22	0.03	5.486	Q2
13	36	0.822	*Journal of Biological Chemistry*	5.486	Q2	*Blood*	1,633	24	0.02	25.476	Q1
14	36	0.822	*Mediators of Inflammation*	4.529	Q3/Q3	*Plos One*	1,594	23	0.00	3.752	Q2
15	33	0.753	*Journal of the American Heart Association*	6.106	Q2	*Nature Reviews Immunology*	1,571	22	0.00	108.555	Q1
16	32	0.730	*Biochemical and Biophysical Research Communications*	3.322	Q3/Q3	*American Journal of Pathology*	1,479	21	0.00	5.770	Q1
17	32	0.730	*Frontiers in Cardiovascular Medicine*	5.846	Q2	*Immunity*	1,479	22	0.00	43.474	Q1
18	32	0.730	*International Journal of Cardiology*	4.039	Q2	*Science*	1,478	22	0.00	63.714	Q1
19	31	0.708	*AIDS*	4.632	Q3/Q2/Q3	*Nature Immunology*	1,446	28	0.06	31.250	Q1
20	31	0.708	*Current Pharmaceutical Design*	3.310	Q3	*Cardiovascular Research*	1,440	25	0.03	13.081	Q1

**Figure 5:**
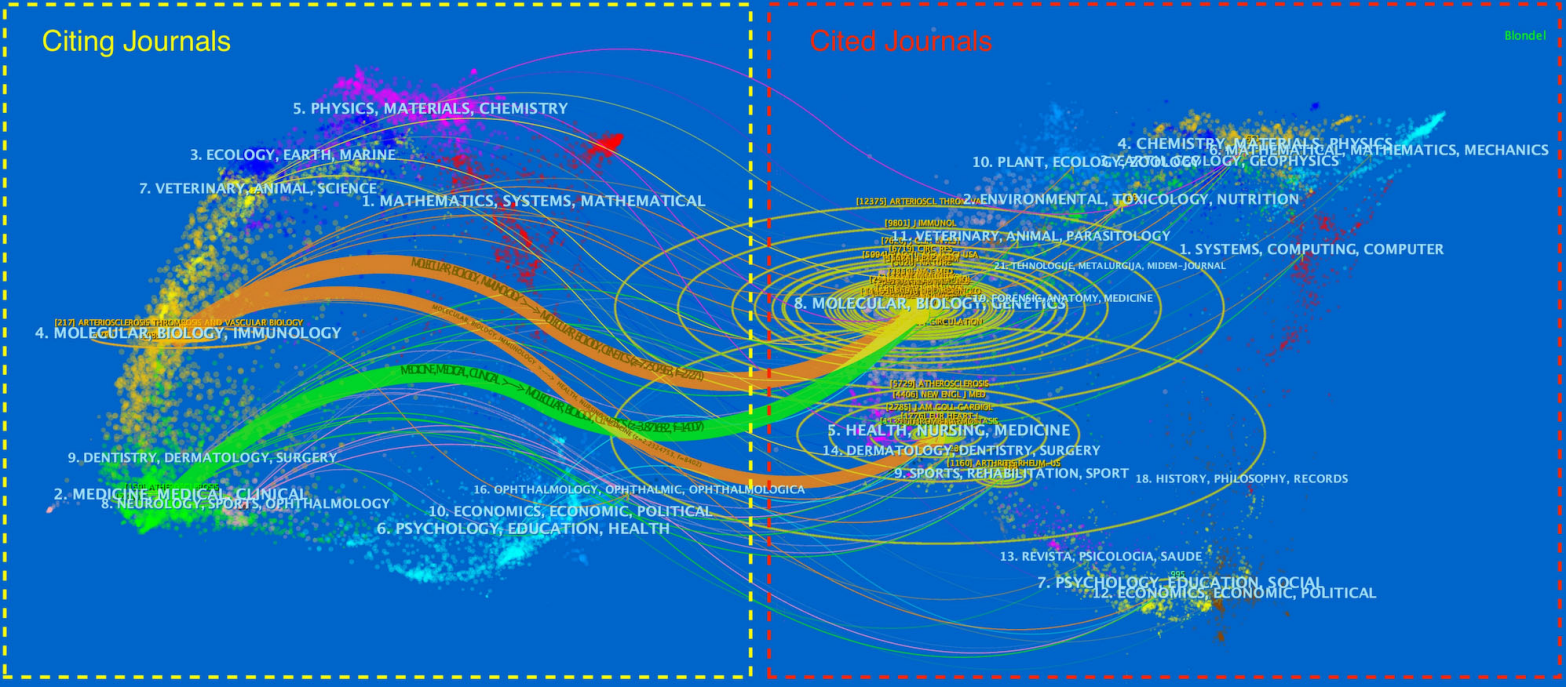
The dual-map overlay of journals in T cell and AS.

In **Figure 5**, dual map overlay of journals shows the distribution of topics. The citing journals are on the left, and the cited journals are on the right. The labels represent the disciplines covered by the journals, and the colored path represented the citation relationship. We can see the most important three paths. The two orange citation paths indicate that research in Molecular, Biology, Genetics journals and Health, Nursing, Medicine journals are frequently cited by Molecular/Biology/Immunology journals. The green citation path indicate that research in Molecular, Biology, Genetics journals is frequently cited by Medicine/Medical/Clinical journals.

### 3.5 Co-cited references and references bursts

**Figure 6:**
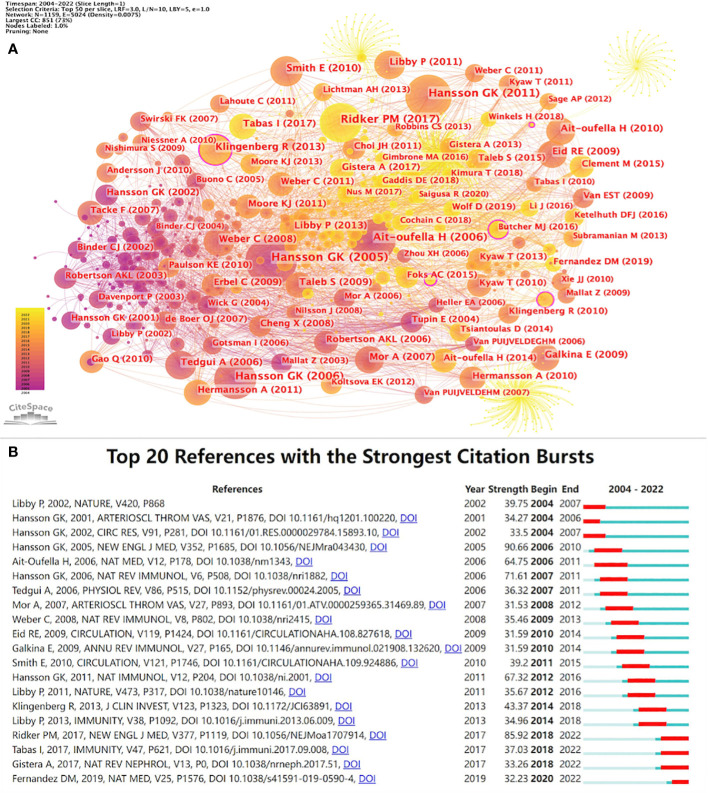
**(A)** References co-citation network in T cell and AS. **(B)** Top 20 references with the strongest citation bursts in T cell and AS.

CiteSpace parameters were set as follows: time slicing (2004–2022), years per slice (1), node type (cited reference), selection criteria (top N=50), and no clipping. According to the set parameters, a network with 1,046 nodes, 4,674 connections, and 0.0086 density was obtained (**Figure 6A**). CiteSpace employs the purple-circled nodes to measure the importance of documents and determine the ten most-cited sources (**Table 7**). Co-citation analysis indicated that two references appeared in the reference list of a third citation article, establishing a co-citation relationship. The article “Inflammation, Atherosclerosis, and Coronary Artery Disease” (29) was the most cited, while “The immune system in atherosclerosis” (30) was the second most cited. The “Antiinflammatory Therapy with Canakinumab for Atherosclerotic Disease” (31) was the third most cited article. In CiteSpace, nodes with more than 0.1 mediation centrality become the key points (32). In **Table 7**, the centrality of “Depletion of FOXP3^+^ regulatory T cells promotes hypercholesterolemia and atherosclerosis” (35) was 0.11. **Figure 6B** presents the top 20 references with the strongest citation bursts. The strongest citation burst was for the 2005 article “Inflammation, Atherosclerosis, and coronary artery disease.” (29). This article has an intensity of 90.66.

**Table 7:** Top 10 cited references of publications in T cell and AS.

Rank	Frequency	Centrality	Title	Journal	Author	Year
1	213	0.00	Inflammation, Atherosclerosis, and Coronary Artery Disease (29)	*N Engl J Med*	Hansson GK	2005
2	182	0.02	The immune system in atherosclerosis (30)	*Nat Immunol*	Hansson GK	2011
3	182	0.02	Antiinflammatory Therapy with Canakinumab for Atherosclerotic Disease (31)	*N Engl J Med*	Ridker PM	2017
4	162	0.02	The immune response in atherosclerosis: a double-edged sword (33)	*Nat Rev Immunol*	Hansson GK	2006
5	162	0.00	Natural regulatory T cells control the development of atherosclerosis in mice (13)	*Nat Med*	Ait-Oufella H	2006
6	102	0.03	Blockade of Interleukin-17A Results in Reduced Atherosclerosis in Apolipoprotein E–Deficient Mice (34)	*Circulation*	Smith E	2010
7	99	0.11	Depletion of FOXP3^+^ regulatory T cells promotes hypercholesterolemia and atherosclerosis (35)	*J Clin Invest*	Klingenberg R	2013
8	90	0.00	Progress and challenges in translating the biology of atherosclerosis (36)	*Nature*	Libby P	2011
9	88	0.03	The multifaceted contributions of leukocyte subsets to atherosclerosis: lessons from mouse models (37)	*Nat Rev Immunol*	Weber C	2008
10	83	0.01	Cytokines in Atherosclerosis: Pathogenic and Regulatory Pathways (38)	*Physiol Rev*	Tedgui A	2006

Specifically, these are representative articles in T cell and AS. The article “Inflammation, Atherosclerosis, and Coronary Artery Disease” (29) is a review by Hansson GK published in *The New England Journal of Medicine* in 2005. This review highlights the importance of inflammation in the pathogenesis of atherosclerotic cardiovascular disease (ASCVD) from four aspects: the main characteristics of atherosclerotic lesions, the evolution of vulnerable plaques, and acute coronary syndrome (ACS), and treatment opportunities.

“The immune system in atherosclerosis” (30); this article is a review published in *Nature Immunology* by Hansson GK and Hermansson A in 2011. This review provides an overview of the role of innate and adaptive immune mechanisms in AS from ten aspects (LDL initiates vascular inflammation; a major role for innate immunity in atherosclerosis; adaptive immunity enters the scene; T lymphocytes: key participants in atherogenesis; role of helper T cell subsets; antigens of atherosclerosis; tolerance, and reactivity to LDL; metabolic regulation of immunity and inflammation; A difficult case for genetic epidemiology; atherosclerosis emphasizes the role of inflammation).

The article “Antiinflammatory Therapy with Canakinumab for Atherosclerotic Disease” (31) was published in *The New England Journal of Medicine* by Ridker PM et al. in 2017. The trial was a randomized, double-blind trial of canakinumab comparing three doses of canakinumab (50, 150, and 300 mg, given subcutaneously every three months) with a placebo. The results revealed that anti-inflammatory treatment with 150 mg canakinumab every three months, targeting the natural IL-1β immune pathway, resulted in a significantly lower recurrence rate of cardiovascular events than placebo, unrelated to reducing blood lipid level.

The centrality of the article “Depletion of FOXP3^+^ regulatory T cells promotes hypercholesterolemia and atherosclerosis” (35) is 0.11, indicating that the article is influential. This article was published by Klingenberg R et al. in *Journal of Clinical Investigation* in 2013. Through the chimeric DEREG/Ldlr^–/–^ mouse model experiment, the authors confirmed that Foxp3^+^ Tregs have a substantial protective effect on AS, and the metabolism of plasma lipoprotein is partially regulated by immune regulation and showed that chronic inflammation could promote cardiovascular diseases (CVDs) by causing metabolic disorder. By mastering the articles with turning significance in this field, we can deeply study the research overview in this field.

### 3.6 Research hotspots and Frontier analysis

As displayed in **Table 8**, in addition to atherosclerosis (2,696) and T cell (1,348), keywords with higher frequency in this study include inflammation (1,348), expression (774), regulatory T cells (699), activation (540), low-density lipoprotein (531), macrophage (469), coronary artery disease (446) and dendritic cells (432). Among these keywords, inflammation, activation, low-density lipoprotein, macrophage, coronary artery disease, and dendritic cells appeared more than 400 times, indicating that they are the focus of research.

**Table 8:** Top 20 keywords in T cell and AS.

Rank	Keywords	Total link strength	Occurrences	Rank	Keywords	Total link strength	Occurrences
1	atherosclerosis	14,037	2,696	11	cardiovascular disease	2,307	409
2	inflammation	8,507	1,508	12	smooth-muscle-cells	2,264	393
3	t cell	7,356	1,348	13	cytokines	2,083	331
4	expression	4,063	774	14	mechanisms	1,889	320
5	regulatory t cells	3,889	699	15	lymphocytes	1,792	296
6	activation	2,926	540	16	myocardial infarction	1,703	284
7	low-density-lipoprotein	3,245	531	17	endothelial cells	1,519	270
8	macrophage	2,728	469	18	c-reactive protein	1,579	255
9	coronary artery disease	2,003	446	19	e-deficient mice	1,581	249
10	dendritic cells	2,453	432	20	immunity	1,388	229

Here includes the automatically calculated total link strength by VOSviewer.

**Figure 7:**
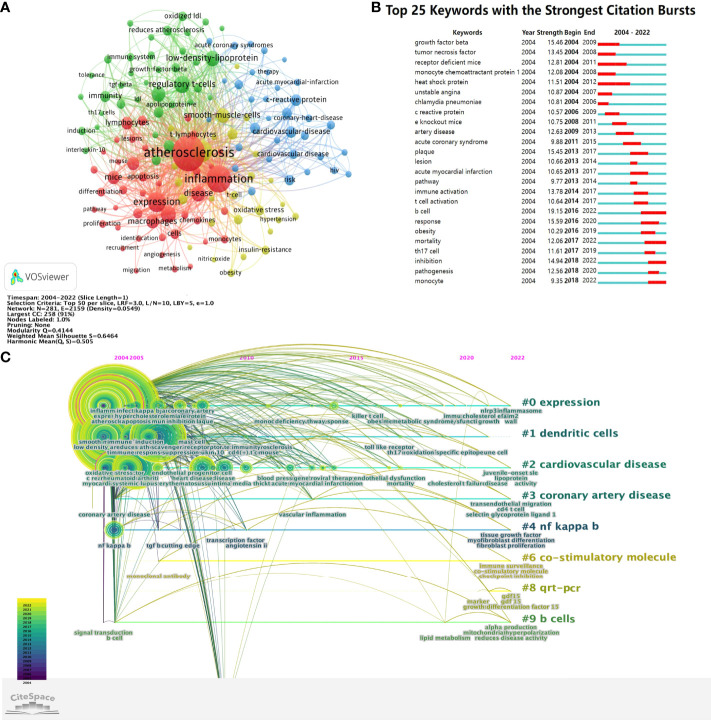
**(A)** The cluster of keywords in the studies of T cell and AS. **(B)** Top 25 keywords with the strongest citation bursts. **(C)** Timeline viewer related to T cell and AS.

VOSviewer parameters were as follows: Method (Linlog/modularity) and a minimum number of occurrences of a keyword: 50. There were 11,731 keywords, with 142 keywords meeting the thresholds. For each of 142 keywords, the total strength of co-occurrence links with other keywords was calculated. Keywords with the greatest total link strength were selected. The keyword co-occurrence network graph (**Figure 7A**) displays that the thicker the connection between nodes, the higher the frequency of two keywords appearing together. Keywords formed four clusters, representing the four main research directions T cell and AS.

The red cluster mainly includes atherosclerosis, inflammation, expression, macrophages, apoptosis, proliferation, angiogenesis, differentiation, monocytes, metabolism, migration, pathway, lymphocytes, t-lymphocytes, atherosclerotic lesions, recruitment, identification, activation, adhesion, atherogenesis, chemokines, cholesterol, deficiency, endothelium, hypercholesterolemia, immune, inhibition, lesions, leukocytes, macrophage, mechanisms, mice, pathogenesis, plaque, progression, protein, receptor, responses, and stains.

The green cluster mainly contains immunity, immune system, low-density-lipoprotein (LDL), IL-10, atherosclerosis reduction, TGF-β, regulatory t-cells (Tregs), apolipoprotein-e, tolerance, Th17 cells, induction, adaptive immunity, antibodies, autoimmunity, B-cells, CD4(+) T-cells, cytokines, deficient mice, dendritic cells, E-deficient mice, e-knockout mice, Foxp3^+^, transforming growth-factor-beta, human atherosclerotic plaques, immune-response, immunization, immunology, *in-vivo*, INF-γ, IL-17, oxidized LDL, receptor-deficient mice, and T cells.

The blue cluster mainly comprises cardiovascular disease, coronary heart disease, artery disease, coronary artery disease, cardiovascular events, heart disease, acute coronary syndrome, acute myocardial infarction, myocardial infarction, unstable angina, rheumatoid arthritis, HIV, stroke, and systemic-lupus-erythematosus.

The yellow cluster mainly consists of smooth muscle cells, endothelial cells, oxidative stress, endothelial dysfunction, gene expression, insulin resistance, T-cell, NO, MCP-1, TNF-α, NF-κB, TLRs, TNF, TNF-α, and INF-γ.

Based on the keywords co-citation network, we conducted the keywords emergent word detection. The top 25 keywords with the strongest citation bursts in T cell and AS are reported. As revealed in **Figure 7B**, the blue line denotes the time axis while the red segment on the blue time axis demonstrates the burst detection, indicating the start year, end year, and burst duration. Notably, B cell (19.150) had the strongest citation bursts, followed by a response (15.590), transforming growth factor-beta (15.460), plaque (15.450), inhibition (14.940), immune activation (13.780), tumor necrosis factor (13.450), receptor-deficient mice (12.810), artery disease (12.630), pathogenesis (12.560), and mortality (12.060). Based on the start time of emergence, it is revealed that transforming growth factor-beta, tumor necrosis factor, receptor-deficient mice, monocyte chemoattractant protein 1, heat shock protein, unstable angina, and chlamydia pneumoniae appeared early and were the subjects of early attention. B cell, mortality, inhibition, and monocyte are the current research frontiers in T cell and AS, and they are already in an explosive period.

Timeline viewer can show the dynamic evolution path of the research hotspots represented by the keywords, and explore the time characteristics of the research fields reflected by the clusters and the rise and fall process of the hotspot keywords research. Documents in the same cluster are placed on the same horizontal line, and the time is from left to right, from far to near. The number of documents in the same cluster highlights the abundance and importance of the research achievements in this cluster field. According to the CiteSpace parameters, a network with 281 nodes, 2,159 connections, and 0.0549 density was obtained (**Figure 7C**). **Figure 7C** intuitively shows the stage hotspots and development directions of T cell and AS research from the time dimension. Cluster ID is the number after clustering. The number is shown as # 0, # 1, # 2, etc. The larger the size of the cluster, the larger the number of members included in the cluster. **Figure 7C** shows eight clusters, which were expression, dendritic cells, cardiovascular disease, coronary artery disease, NF kappa b, co-stimulatory molecule, qrt-pcr, and b cells. From **Figure 7C**, we can see that the keywords involved in 2004 and 2005 are mainly inflammation, coronary artery disease, NF kappa b, singal transduction, b cell. The keywords involved in 2020-2022 are mainly NLRP3 inflammasome, toll-like receptors, transendothelial migration, cd4 t cell, selection glycoprotein ligand 1, fibroblast proliferation, mitochondrial hyperpolarization, checkpoint inhibition factor 15.

## 4 Discussion

### 4.1 General information

In this study, we analyzed T cell and AS documents and reviewed the research results and progress using CiteSpace and VOSviewer quantitative analysis software. The basic information of annual publication quantity, country, author, institution, discipline, and journal is quantitatively analyzed. According to the number of documents issued by T cell and AS, the number of documents published in this field from 2004 was 144, and the overall trend is increasing. The higher citations a paper has, the greater its impact on the field and the higher its quality. As **Figure 2** displays, the number of citations in this field has annually increased. Based on the statistical analysis of the number of papers published by various countries/regions and institutions, it is possible to identify key countries/regions and research institutions that have published many T cell and AS documents and have greater influence and determine their cooperative relationship. USA, China, and Germany are the countries that mainly study T cell and AS. Sweden, USA, and Australia have relatively mature studies in T cell and AS. Among the top ten institutions, three are from the Netherlands, two are from the USA, and two are from France, with Harvard University being the institution with the most publications and the highest h-index. The cooperation between various countries and institutions is relatively close. Close cooperation and communication between countries and institutions are conducive to eliminating academic barriers and further developing T cell- and AS-related research.

From the top 10 authors, Hansson GK (97, 2.214%) is the most published author, followed by Weber C (78, 1.780%) and Nilsson J (70, 1.598%). It indicates that these three authors have made the most outstanding contribution in the field of T cell and AS. Professor Hansson GK from the Department of Medicine, Center for Molecular Medicine (G.K.H.) is the author with the highest h-index (39). H-index is a comprehensive quantitative index used to evaluate the quantity and level of academic output of researchers (40). Prof. Hansson GK reviewed the immune system’s role in AS and believed that immune response in AS is a double-edged sword (30, 33). Experimental and clinical knowledge of AS pathogenesis is elucidated from an immunological perspective (41, 42), and new treatments are sought (43). This was followed by Weber C from IPEK and Nilsson J from Department of Clinical Sciences Malmö, Lund University, Sweden. Weber C studied the critical role of chemokines in AS (44) and suggested that AS is a chronic inflammatory disease of arterial walls caused by chemokine-driven monocyte recruitment (45, 46). As small chemotactic cytokines, chemokines are key mediators and regulators of leukocyte trafficking during immune surveillance and inflammation (47, 48). Nilsson J has studied many important roles of regulatory T cells in AS, inhibiting autoimmunity development by controlling the activity of autoreactive T cells (49). The response of regulatory T cells to apolipoprotein B100-derived peptides reduces AS development and progression in mice (50, 51), and vaccination may be an effective strategy to prevent or regulate AS (52, 53). There are no authors whose centrality is greater than or equal to 0.10 among all authors, indicating that there are no authors with significant influence in this field.

According to the discipline distribution in **Table 5**, the discipline with the most articles about T cell and AS is Cardiac Cardiovascular Systems (967, 22.073%), Peripheral Vascular Disease (922, 21.045%), and Immunology (812, 18.535%). According to journal distribution in **Table 6**, the journals with the most articles on T cell and AS are *Arteriosclerosis Thrombosis and Vascular Biology* (243, 5.547%), followed by *Atherosclerosis* (179, 4.086%) and *Plos One* (139, 3.173%). *Circulation* and *Arteriosclerosis Thrombosis and Vascular Biology* were frequently co-cited. Among the top 20 in co-citing journals, 9 journals are located in Q1 JCR region, of which *Circulation* (39.918, Q1/Q1) has the highest IF. Among the top 20 in co-cited journals, 17 journals are in the JCR1 region, and 15 journals have IF greater than 10. The journal with the highest IF is *New England Journal of Medicine* (176.079). JCR profiles are journal-specific indicators related to impact factors, rankings, and quarters in categories (39). Analyzing the distribution of literature sources, it is helpful to find the core journals published in the relevant literature on T cell and AS and help scholars build scientific achievements. These indicate that many high quality and high impact journals are very interested in T cell and AS related research. These data will help future scholars select journals when submitting manuscripts related to T cell and AS. **Figure 5** shows that papers published in Molecular, Biology, Genetics journals and Health, Nursing, Medicine journals are frequently cited in papers published in Molecular, Biology, Genetics journals and Medicine, Medical, Clinical journals. This means that current research on T cell and AS-related research is mainly focused on basic research and transitional medicine.

Most of the top ten references dealt with immune responses and inflammation, mainly because AS was previously thought to be due to the passive accumulation of cholesterol in the arterial wall (54). With increasing evidence that AS was gradually recognized as a chronic inflammatory disease in the late 1990s, innate and adaptive immune-inflammatory mechanisms are involved in all disease stages, in which T cells are critical in the immune response accompanying AS (55). The high burst signal of reference indicates the high intensity of interest and time interval (56). According to the 20 references with the strongest citation bursts, the most cited references in recent years is mainly related to the immune and inflammatory mechanism of AS. It was not until around 2000 that people realized that the inflammatory mechanism combined dyslipidemia with AS formation, and the dysfunction of inflammatory mediators inhibited AS formation in mice (54).

### 4.2 Hotspots and Frontiers

Analysis of high-frequency keywords reflects the hotspots in a particular research field. We used key co-occurrence analysis to determine the main directions and hotspots in T cell and AS, as well as to uncover the development and changes of its theme structure (57). Cluster analysis based on keywords, finally forming a cluster of four colors. Then, according to the analysis of the top 25 keywords with the strongest citation bursts, the research hotspots and frontiers in T cell and AS are determined, and its main contents are as follows:

#### 4.2.1 Inflammatory mechanism of AS

According to keywords, the red cluster mainly clarifies the inflammatory mechanism of AS. It is well known that AS is a chronic inflammatory lesion of large and medium elastic arteries in the whole body (58), and inflammatory reaction runs through the whole process of AS occurrence and development (59). The study on the results of Canakinumab anti-inflammatory thrombosis (31), the cardiovascular inflammation reduction trial of non-specific anti-inflammatory treatment with methotrexate (60), and the cardiovascular outcome test of colchicine (61, 62) all exhibited anti-inflammatory effects. The potential value of inflammation in AS and coronary heart disease treatment. Currently, targeted therapeutic drugs for inflammatory factors have made preliminary progress, such as IL-1β; IL-6 is the most potential target for inflammatory targeted therapy of AS. By studying NF-κB, JAK/STAT, and other AS-related inflammatory signaling pathways can further understand AS pathogenesis. Studies have also revealed that powerful immunosuppressants or anti-inflammatory drugs may be attractive approaches for treating acute coronary syndromes, demonstrating that strategies to reduce inflammation may be a viable treatment for AS suppression (63). However, the role of inflammation in AS pathophysiology is complex, and the interaction mechanism between metabolic imbalance and immunomodulatory disorder continues to be further researched; thus, it is also the focus of current research.

#### 4.2.2 Immune mechanism of AS

The second cluster is mainly associated with the immune mechanism of AS. AS is the main pathological basis of ischemic heart, cerebrovascular and peripheral vascular diseases. The immune response is involved in each stage of AS—the formation of atherosclerotic plaques, the transformation from stable to unstable plaques, and the rupture of plaques (64). There is increasing evidence that innate and adaptive immunities can closely regulate AS occurrence (65). The innate immune response is the first line of defense of immune response and the basis of adaptive immune response. It mainly plays the role of scavenging damaged and apoptotic cells, antigen presentation, immunosuppression, and protecting the host from foreign microorganisms (66). Adaptive immune response, the center of immune memory and tolerance development, can recognize presented antigens and release antibodies (62). The main feature of AS is that endothelial injury releases adhesion molecules, triggers innate immune response dominated by monocytes and macrophages, and then dendritic cells and macrophages present antigens to cause adaptive immune response dominated by lymphocytes. The inflammatory mechanism of AS is based on numerous immunoactivity cells found in AS lesions, such AS congenital immune cells (monocytes, macrophages, dendritic cells, etc.), adaptive immune cells (T cells, B cells, etc.), and various cytokines produced. Examples include IL, tumor necrosis factor (TNF), interferon (IFN), transforming growth factor (TGF), colony-stimulating factor (CSF), and chemokines (67). Among them, pro-inflammatory cytokines such as TNF-α, IL-1β, monocyte chemoattractant protein-1 (MCP-1), IL-6, IL-1, IL-12, and type I IFN (IFN-γ, β) and accelerate AS progression; Anti-inflammatory cytokines such as IL-10 and TGF-β, Arginase-1 (Arg-1), forkhead or winged-helix transcription factor-3(FOXP3) and IL-5 improved AS development. In addition, antibodies secreted by immune cells (immunoglobulin E (IgE), IgG, and IgM) and other specific binding-related antigens (heat shock protein (HSP) and oxidized low-density lipoprotein (ox-LDL)) play an immunomodulatory role. Studies have revealed that blocking TGF-β signaling pathway in ApoE^-/-^ mouse T cells promote AS (68). Therefore, AS occurrence and development can be delayed, reduced, or even reversed by further regulation of immune regulatory cells and cytokines from the perspective of inflammation and interruption of key targets of immune-inflammatory response (69).

In addition, the cluster involves model animals for studying AS. Clinical studies, population studies, and cell culture experiments have provided important clues to AS pathogenesis (70). However, experiments on animals are required to dissect the pathogenic steps and determine cause and effect. Under normal conditions, laboratory mice do not develop AS.

However, ApoE^-/-^ mouse causes severe hypercholesterolemia and spontaneous AS. LDL^-/-^ mouse also develops AS, especially in mice fed a high-fat diet. Generally, these knockout mice have been used to study the relationship between hypercholesterolemia and AS and evaluate the impact of other genes and gene products on these diseases. By mating these mice with knockout mice lacking immunoregulatory genes, it is possible to elucidate the role of immune and inflammatory mechanisms in AS (29). In summary, the immune system is critical in AS occurrence and development, and more immunotherapy strategies will be applied to treat AS-related diseases in the future. Further improving the theory of AS and the prognosis of patients with related diseases is one of the hotspots of current research.

#### 4.2.3 AS-related diseases

The blue cluster mainly involves AS-related diseases. AS, the underlying pathological basis of cardiovascular and cerebrovascular diseases, is prevalent in the elderly, and its incidence increases with age, dominating global mortality and disability statistics (3). The pathological process of AS progresses slowly. It mainly demonstrates clinical symptoms when the coronary lumen is seriously narrowed, and acute thrombosis seriously blocks the blood flow. Blocking blood flow to the heart will lead to coronary heart disease, blocking blood flow to the brain will cause ischemic stroke, and reducing blood flow at the end of the limb will result in peripheral vascular disease. AS is prevalent in the elderly, and its incidence increases with age. In addition, studies have revealed that premature AS is one of the main reasons that affect the quality of life of patients with systemic lupus erythematosus (SLE) and leading to death in SLE patients, and there is an increasing trend (71). The increased prevalence of AS in SLE patients is thought to be due to a combination of traditional risk factors and autoimmune-mediated inflammatory responses (72).

#### 4.2.4 AS-related cytokines

The yellow cluster mainly involves AS-related cytokines. Various cytokines and related substances are involved in AS occurrence and development (73) and are critical in AS occurrence and development. Cytokines are a very diverse class of molecules, including more than 100 secretory factors, which can be divided into several categories: ILs, tumor necrosis factors (TNFs), interferons (IFNs), transforming growth factors (TGFs), colonies Stimulatory factor (CSF), and various chemokines (CXCL). The expression of cytokines can be abnormal before morphological changes of AS. Therefore, detecting cytokines can provide more effective information for discovery, diagnosis, and curative effect evaluation of AS-related diseases. Studies have revealed that most communication between monocytes/macrophages and vascular smooth muscle cells is through immune mediators (74), with TNF-α, IL-1β, IL-6, and MCP-1 in AS and plays a major role in arterial wall inflammation (75). With the deepening and development of research, inflammatory factors are critical in AS-related diseases. However, discovering new AS-associated cytokines and the related mechanisms remains challenging.

#### 4.2.5 B cell, mortality, inhibition, and monocyte are the frontiers of research in this field and are currently in an explosive phase

##### 4.2.5.1 B cell

The first research frontier may be B cell. It is well known that the proliferation and activation status of B cells, in addition to T cells, are critical factors in CVD risk. B cells are involved in the systemic and local immune responses of AS arteries through antigen presentation, cytokine production, and immunoglobulin synthesis (76, 77). The specific relationship between B cells and AS depends largely on the subset of B cells and their functional targeting (78, 79). B cells were divided into B1 and B2 subsets. In mature B cells, B2 cells dominated.

B1 cells assume the natural defense function against common pathogens. Accumulating IgM antibodies derived from B1 cells in AS lesions can reduce the number of apoptotic cells and the necrotic core size to reduce the severity of inflammation and block the uptake of ox-LDL by macrophages (80), which has a protective effect on AS. Studies have indicated that transplantation of B cell-deficient bone marrow into irradiated LDLR^-/-^ mice exacerbates AS (81). Subsequent studies further confirmed that the lack of B1a cells and the consequent decrease in IgM are the leading causes of the loss of B cell effect (82). This study also confirmed that IgM released by B1a cells protects the vascular wall by inhibiting necrotic core formation. In addition, studies have demonstrated that IgM inhibits ox-LDL uptake by macrophages, thereby inhibiting myeloid cell inflammation (83). Similarly, the response of IgM secreted by B1b cells to oxidation-specific epitopes also has a protective effect on AS (84). B2 cells are further divided into marginal zone B cells and follicular B cells. Marginal zone B cells play an anti-AS function, while follicular B cells aggravate AS (85, 86). Inhibiting B2 cells is protective against AS (87, 88).

In addition, there are regulatory B cells (Bregs), accounting for only a small part of B cells, but can still inhibit vascular inflammation by producing anti-inflammatory cytokine IL-10 to produce the effect of anti-AS (89).

In conclusion, the complex role of B cells in AS has not yet been fully and accurately understood. Therefore, it is very necessary to further explore the mechanism of B cells regulating AS to gain a more comprehensive understanding of AS. Simultaneously, B cell-targeted therapy of AS is an important research direction in the future.

##### 4.2.5.2 Mortality

The second research frontier may be mortality. Cardiovascular and cerebrovascular diseases caused by AS are now the leading cause of death worldwide, and morbidity and mortality are rising (90). Therefore, the related understanding of mortality will still be future research hotspot and frontier.

##### 4.2.5.3 Inhibition

The third research frontier may be inhibition. The wide clinical application of immune checkpoint inhibitors (ICIs) increases the risk of immune-related adverse events in the cardiovascular system. In cancer patients, the adverse effects of ICIs on AS-related cardiovascular disease may lead to acute cardiovascular events (AVEs) such as myocardial infarction (91). Therefore, conducting predictive studies to increase clinician awareness of AVEs caused by ICIs through risk stratification is essential in preventing these adverse events.

##### 4.2.5.4 Monocytes

The fourth research frontier may be monocytes. Monocytes are an important part of the body’s innate immune system and the leukocytes recruited at the early stage of AS lesions, which can further differentiate into macrophages to promote inflammation. Macrophages phagocytose ox-LDL accumulation in the vascular wall to form foam cells that initiate necrotic core formation in the plaque, further exacerbating atherosclerotic lesions. At present, human monocytes can be divided into three groups according to the expression of surface markers CD14 and CD16: classical (CD14++CD16-), intermediate (CD14++CD16+), and non-classical (CD14+CD16++) monocytes (92). Monocytes in mouse circulating blood include two main subtypes: Ly6C(hi) and Ly6C(low). Studies have revealed that hypercholesterolemia can trigger monocyte increase in mice. Reversing hypercholesterolemia in mice can prevent monocyte recruitment and reduce macrophages in plaques, suggesting that continuous monocyte recruitment is necessary for plaque maintenance and progress (93). The capture and rolling of circulating monocytes depend on a series of chemokines expressed by vascular endothelial cells, such as CCL5, CXCL1, and P-selectin. The entry of monocytes from the endothelium into AS plaques requires the participation of chemokines secreted by endothelial cells, macrophages, and smooth muscle cells. The most important three pairs of chemokine receptors-chemokines are CCR2-CCL2, CX3CR1-CX3CRL1, and CCR5-CCL5 (94). Combined inhibition of these three chemokine axes could prevent the increase of monocytes in hyperlipidemic mice and reduce the area of AS plaque in ApoE^-/-^ mice by approximately 90% (95).

In summary, B cell, mortality, inhibition, and monocyte are the frontiers of research in this field and are currently in an explosive phase.

### 4.3 Limitations

First, to ensure high-quality bibliometric analysis, the analysis of this study is based on the articles in WoSCC database, which is the most famous database of scientific publications on many research topics. Numerous studies have been omitted because they are published in non-SCI journals or other databases. Second, CiteSpace and VOSviewer cannot completely replace system retrieval. Third, bibliometrics cannot assess the quality of individual studies because citation metrics are time-dependent, implying that more recent articles may be less cited than earlier articles, mainly due to publication date (96). These limitations may slightly impact the overall results but are unlikely to change the main trends presented in this paper. Overall, our study provides a foundation for understanding research topics, hotspots, and development trends in T cell and AS.

## 5 Conclusion

Through detailed bibliometric analysis in T cell and AS, this study evaluates the literature information of different years, countries, institutions, authors, disciplines, and journals and analyzes the theme development and future research hotspots. Our study observed that the field began to garner attention in 2004. Our study provides basic information about research in this field and identifies potential partners for interested researchers. Current research hotspots mainly include the inflammatory mechanism, and immune mechanisms, related diseases, and related cytokines of AS. Currently, B cell, mortality, inhibition, and monocyte are the frontiers of research in this field and are currently emerging.

## Data availability statement

The original contributions presented in the study are included in the article/supplementary material. Further inquiries can be directed to the corresponding authors.

## Author contributions

NW and YX designed the study. YL, JS, and XZ collected the data. NW, YX, and YY re-examined the data. NW, YX, and QS analyzed the data. NW and YX wrote the manuscript. YH and HZ reviewed and revised the manuscript. All authors contributed to the article and approved the submitted version.

## Acknowledgments

The authors thank Home for Researchers editorial team (www.home-for-researchers.com) for language editing service.

## Conflict of interest

The authors declare that the research was conducted in the absence of any commercial or financial relationships that could be construed as a potential conflict of interest.

## Publisher’s note

All claims expressed in this article are solely those of the authors and do not necessarily represent those of their affiliated organizations, or those of the publisher, the editors and the reviewers. Any product that may be evaluated in this article, or claim that may be made by its manufacturer, is not guaranteed or endorsed by the publisher.
